# The Coadministration of N-Acetylcysteine Ameliorates the Effects of Arsenic Trioxide on the Male Mouse Genital System

**DOI:** 10.1155/2016/4257498

**Published:** 2015-12-29

**Authors:** Raquel Frenedoso da Silva, Cibele dos Santos Borges, Patrícia Villela e Silva, Gabriela Missassi, Luiz Ricardo Almeida Kiguti, André Sampaio Pupo, Fernando Barbosa Junior, Janete Aparecida Anselmo-Franci, Wilma De Grava Kempinas

**Affiliations:** ^1^Department of Morphology, Institute of Biosciences, Universidade Estadual Paulista (UNESP), 18618-970 Botucatu, SP, Brazil; ^2^Department of Pharmacology, Institute of Biosciences, Universidade Estadual Paulista (UNESP), 18618-970 Botucatu, SP, Brazil; ^3^Department of Clinical Analyses, Toxicology and Food Sciences, School of Pharmaceutical Sciences, University of São Paulo (USP), 14040-903 Ribeirão Preto, SP, Brazil; ^4^Department of Morphology, Stomatology, and Physiology, School of Dentistry, University of São Paulo (USP), 14040-903 Ribeirão Preto, SP, Brazil

## Abstract

Arsenic trioxide (As_2_O_3_) has shown effectiveness in treatment of leukemia but is also associated with reproductive toxicity. Since remediation with N-acetylcysteine (NAC) may mitigate the adverse effects caused by exposure, we assessed the effects of As_2_O_3_ and its potential reversibility after exposure cessation or coadministration of NAC. Animals received 0.3 or 3.0 mg/Kg/day of As_2_O_3_ subcutaneously and 40 mM of NAC in tap water. As_2_O_3_ treatment impaired spermatogenesis and sperm motility and decreased seminal vesicle weight and testosterone serum levels; after suspension of treatment, these parameters remained altered. When NAC was administered, animals showed improvement in sperm parameters and seminal vesicle weight. *In vitro* epididymal contractility was increased in As_2_O_3_-treated animals. We concluded that As_2_O_3_ is toxic to the male mouse genital system by compromising sperm quality and quantity; these effects persisted even after suspension of the treatment. However, the coadministration of NAC ameliorates the harmful effects of the drug on the male genital system.

## 1. Introduction

Arsenic is a heavy metal that is widespread in the soil, water, air, and organisms. The consequent inevitability of natural exposure to this metal characterizes it as an environmental contaminant with known carcinogenic, mutagenic, and teratogenic effects [1]. This metal occurs in two oxidation states: the trivalent form, arsenic trioxide or arsenite (As_2_O_3_), and the quinquivalent form, arsenate (As_2_O_5_), with the former state being 60 times more toxic than the latter [2].

In 1970, a research group from China found that intravenous injections of a solution containing As_2_O_3_ could be used in the treatment of acute promyelocytic leukemia (APL) and were able to promote complete disease remission in over 60% of the cases [3]. Ever since, several studies have confirmed the role of this compound in inducing complete remission in over 80% of leukemia patients undergoing treatment [4–8].

The drug acts through induction of differentiation and programmed cell death of malignant cells [9]. Despite its efficiency in the treatment of leukemia, As_2_O_3_ can be toxic to the male genital system, since it can cause apoptosis in germ cells. Studies showed that As_2_O_3_ can induce Sertoli cell apoptosis and decrease testosterone levels and sperm motility [10, 11]. However, it is unknown whether these effects on the genital system persist when exposure ends.

N-acetylcysteine (NAC) is an antioxidant substance that plays an important role in the protection of cell constituents from oxidative stress. Since the toxicity of As_2_O_3_ is closely related to the generation of reactive oxygen species (ROS), a process that leads to lipid peroxidation and DNA damage in exposed cells [12], remediation with NAC can possibly mitigate the adverse effects caused by As_2_O_3_. Thus, the present study aimed at assessing the effects of As_2_O_3_ on male mouse genital system based on epididymal duct contractility and sperm parameters, besides their potential reversibility after either the cessation of exposure or coadministration of NAC.

## 2. Material and Methods

### 2.1. Animals

Swiss male mice (70 days old, weighing 30–40 g) were supplied by the Multidisciplinary Center for Biological Investigation, State University of Campinas. Animals were kept in a controlled environment with temperature at ±23°C, humidity of 55 ± 5%, and 12-h light/dark cycle (lights on 7:00 a.m.) and had free access to regular lab chow and tap water. The experimental procedures were approved by the Ethics Committee for the Use of Experimental Animals from Universidade Estadual Paulista (UNESP), Botucatu (protocol number 429-CEUA), and are in accordance with the Guide for the Care and Use of Laboratory Animals (National Institutes of Health).

### 2.2. Drugs and Solutions

As_2_O_3_ was obtained from Acros (1327-53-3, arsenic(III) oxide, 99.5%) and* N*-acetyl-L-cysteine, norepinephrine (NE) (NE bitartrate salt monohydrate), and carbachol (CCh) (carbamylcholine chloride) were obtained from Sigma-Aldrich, St. Louis, MO, USA.

**Experiment 1 (assessment of As_2_O_3_ effects in male genital system and their reversibility after a recovery period).**

Animals were randomly allocated into three experimental groups: control group treated with distilled water (vehicle) and treated groups that received 0.3 and 3.0 mg/Kg/day of As_2_O_3_ (*n* = 20/group, diluted in the vehicle), subcutaneously, administered 5 days per week, followed by 2 days of interruption, for 5 weeks. Treatment regimen and dose selection were based on consolidation therapy used for humans. According to Soignet et al. [4], As_2_O_3_ was effective in APL treatment at doses of 0.06 to 0.2 mg/Kg, above which no difference in effectiveness was noted. Since there is a correspondence between the appropriate doses for humans and those used in animal studies, it is necessary to normalize for body surface area (BSA), as previously described [13]. The higher dose proposed in this study is equivalent to a dose of 0.2 mg/Kg for humans.

After the last administration of As_2_O_3_, mice from the control and treated groups (10 animals per group, 105 days old) were weighed and euthanized by cervical dislocation to evaluate the immediate effects of the treatment on the male genital system. Furthermore, the other 10 animals of each group were kept without treatment for a period of 50 days (receiving just normal water and food), approximately corresponding to the time of spermatogenesis and sperm transit through epididymis [14]. After this period, 155-day-old mice were euthanized in order to assess the potential reversibility of the effects.

### 2.3. Organ Weights

Immediately after the euthanasia, the kidney, liver, testis, epididymis, ventral prostate, and seminal vesicle (without the coagulating gland) were removed and had their wet weights (absolute and relative to body weight) measured.

### 2.4. Testosterone Measurement

After euthanasia, blood was collected by cardiac puncture (between 9:00 and 11:30 a.m.) and serum was obtained by centrifugation (1236 ×g, for 20 min at 4°C). The testosterone concentrations were determined by double antibody radioimmunoassay, using a TESTOSTERONE MAIA kit (Biochem Immuno System).

### 2.5. Arsenic Measurements in Blood

For the determination of arsenic in whole blood, coupled plasma mass spectrometry (ICP-MS) was used. Reagents and solutions were as follows: high-purity deionized water (resistivity 18.2 MW·cm) obtained by the Milli-Q system (Millipore) was used throughout the analysis. Nitric acid was distilled at a temperature lower than the boiling point, using a Kürner Analysentechnik Quartz retort to eliminate impurities. All solutions were stored in polyethylene bottles. Plastic bottles, auto sampler cups, and glassware were immersed in a solution containing 20% v/v HNO_3_ for 24 h, washed five times with Milli-Q water, and dried in a laminar flow class 100. All operations for the preparation of standard solutions of arsenic were conducted in a clean room class 10000. The internal standard was diluted from 1000 mgL^−1^ stock standard (PerkinElmer, Norwalk, CT, USA), standardized by the National Institutes of Standards and Technology (NIST).

### 2.6. Sperm Motility

Sperm were obtained from the right vas deferens and immediately diluted in 0.3 mL modified HTF medium (Human Tubular Fluid, Irvine Scientific), prewarmed to 34°C. Then an aliquot of 10 *μ*L of the diluted sperm was placed in a Makler chamber (Irvine, Israel) and analyzed under a light microscope at 400x magnification. One hundred sperm were evaluated per animal and classified for motility into type A, motile with progressive trajectory, type B, motile with nonprogressive trajectory, or type C, immotile.

### 2.7. Daily Sperm Production per Testis

The right testis was used for spermatid counts. Homogenization-resistant testicular spermatids (stage 16 of spermiogenesis) in the testis were counted as described previously [15]. Briefly, the testis was decapsulated, weighed, and homogenized in 5 mL NaCl 0.9% containing Triton ×100 at 0.5%, followed by sonication for 30 s. After a 10-fold dilution, one sample was transferred to Neubauer chambers (five fields per animal), and late spermatids were counted. To calculate the daily sperm production (DSP), the number of homogenization-resistant spermatids was divided by 4.84, the number of days that these spermatids are present in the seminiferous epithelium.

**Experiment 2 (coadministration of As_2_O_3_ and antioxidant NAC).**

In this step of the experiment, Swiss male mice (70 days old) were divided into four groups (*n* = 8 per group): control group, treated with distilled water subcutaneously; As_2_O_3_ group, which received 3.0 mg/Kg/day of As_2_O_3_ subcutaneously, diluted in the distilled water; NAC group, treated with distilled water subcutaneously plus 40 mM of NAC in tap water [16]; and As_2_O_3_ + NAC group, which received 3.0 mg/Kg/day of As_2_O_3_ (diluted in distilled water) plus 40 mM of NAC in tap water. Animals were treated 5 days per week, followed by 2 days of interruption, for 5 weeks, as previously described in Experiment 1; NAC was given to animals for 5 weeks, without interruption. At the end of treatment, animals were euthanized by cervical dislocation for evaluation of the protective effects of NAC on seminal vesicle weight, sperm motility, and sperm count and concentration, that is, parameters before being affected by exposure to As_2_O_3_, following the methodology described above.

**Experiment 3 (pharmacological assay).**

Since As_2_O_3_ is involved in modifications of intracellular calcium, exposure to this substance can increase contractility of smooth muscle surrounding the cauda epididymis that can accelerate sperm transit through the epididymis. These modifications on transit time can compromise potential fertility. Therefore we decided to evaluate epididymal duct contractility of both* in vivo* treated animals and* in vitro* As_2_O_3_-exposed tissue.

### 2.8. *In Vitro* Tension of the Isolated Epididymal Duct of Animals Treated with Vehicle and As_2_O_3_

The cauda epididymis (CE) duct was used for evaluation of the effects of* in vivo* treatment with As_2_O_3_ on the tension. After completion of treatment (control group (*n* = 10), which received vehicle, and As_2_O_3_ group (*n* = 10), which received 3.0 mg/Kg/day of As_2_O_3_, as in Experiment 1) male mice were euthanized by cervical dislocation and the left epididymis was isolated. The epididymal duct from the distal cauda was uncoiled with scissors and the luminal contents were washed with nutrient solution (composition below). A CE duct segment approximately 1.0 cm in length was isolated and attached to a FORT10 isometric force transducer (WPI, USA), connected to a Transbridge 4M Transducer Amplifier (WPI, USA), to record the isometric tension development in a PC-based System (MP100, Biopac System Inc., USA) and analyzed offline using the software AcqKnowledge version 3.5.7 (Biopac System Inc., USA) (please see Figure S1 in Supplementary Material available online at http://dx.doi.org/10.1155/2016/4257498). CE ducts were mounted under a 0.5 g resting tension and maintained in a modified Tyrode's solution of the following composition (mM): NaCl 138; KCl 5.7; CaCl_2_ 1.8; NaH_2_PO_4_ 0.36; NaHCO_3_ 15; dextrose 5.5, prepared in glass-distilled deionized water, pH 7.4, maintained at 30°C and continuously bubbled with 95% O_2_/5% CO_2_. After mounting, a 30-minute stabilization period was allowed.

After the stabilization period, the tension developed for 80 mM KCl was evaluated twice at 30-minute interval to ascertain tissue viability. The CE duct tension from each mouse developed for 80 mM KCl, and NE (10^−8^ M–10^−4^ M) and the muscarinic receptor agonist CCh (10^−6^ M–10^−3^ M) were recorded. The maximal tension developed (*E*_max⁡_, in grams of tension, g) and the potency of NE and CCh in inducing epididymal duct tension (expressed as pEC_50_ the −log of NE and CCh concentration inducing 50% of maximal tension) were evaluated.

### 2.9. *In Vitro* Tension of the Isolated Epididymal Duct of Untreated Animals Exposed to As_2_O_3_ *In Vitro*

Untreated mice were killed by cervical dislocation; both epididymides were collected and the distal CE duct (1 segment per epididymis) was isolated and mounted as described above. From the same animal, one CE duct was exposed* in vitro* to As_2_O_3_ and the other was maintained as the untreated control (without drug exposure). Treated CE ducts were exposed to different concentrations of As_2_O_3_: 10 *μ*M for 45 minutes, 10 *μ*M for 2 hours, and 100 *μ*M for 2 hours and then a cumulative concentration-response curve for NE (10^−8^ M–10^−4^ M) was obtained. Control tissues were maintained untreated for the same time period as the treated CE ducts and then a concentration-response curve for NE was obtained. The maximal tension and the pEC_50_ induced by NE were evaluated.

### 2.10. Statistical Analysis

Data are presented as mean ± Standard Error of Mean (SEM) or median and interquartile range. Parametric variables were compared by ANOVA followed by the test of Tukey or Newman-Keuls, and nonparametric variables were compared by Kruskal-Wallis followed by Dunn's test. Differences were considered significant when *p* < 0.05. The statistical analyses were performed by the software GraphPad Prism (version 5.0).

## 3. Results

**Figure 1:**
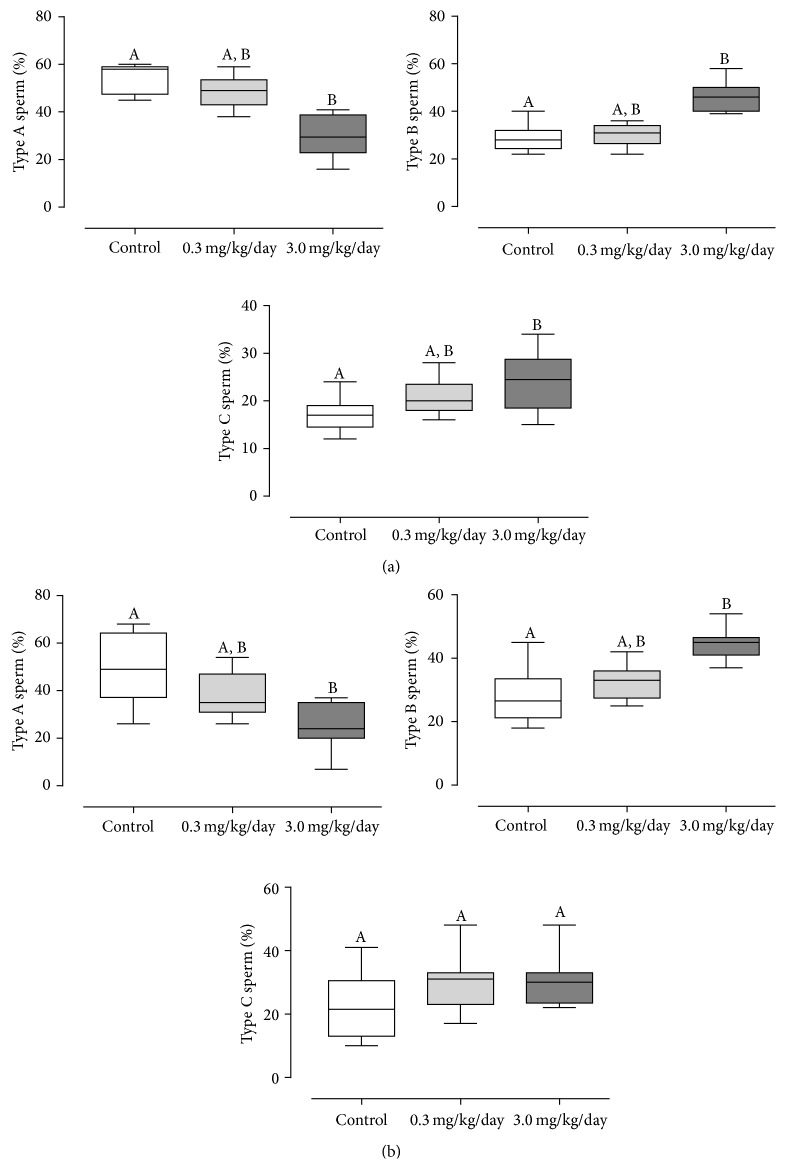
Sperm motility in (a) 105-day-old (*n* = 10 per group) and (b) 155-day-old (*n* = 10 per group) animals. Values expressed as median and interquartile range. Kruskal-Wallis analysis of variance test, followed by Dunn's test. Different letters indicate medians that differ at 5% significance level.

Body weight gain was similar among experimental groups (data not shown). However, at the end of treatment, absolute and relative full seminal vesicle weights were significantly decreased in the group treated with 3.0 mg/Kg/day of As_2_O_3_. After the recovery period, this same group showed increased liver and kidney relative weights and continued to show reduced full seminal vesicle weight (Table 1). Assessment of sperm quality revealed statistically significant reduction in type A sperm in the highest dose group; consequently, the percentage of type B sperm and type C sperm was significantly increased. After suspension of treatment, this group continued presenting diminution in percentage of type A sperm and elevation in type B sperm (Figure 1). Furthermore, evaluation of sperm counts both after the treatment and after recovery period showed that treatment with 3.0 mg/Kg/day of As_2_O_3_ reduced testicular sperm counts and DSP (Table 2).

**Figure 2:**
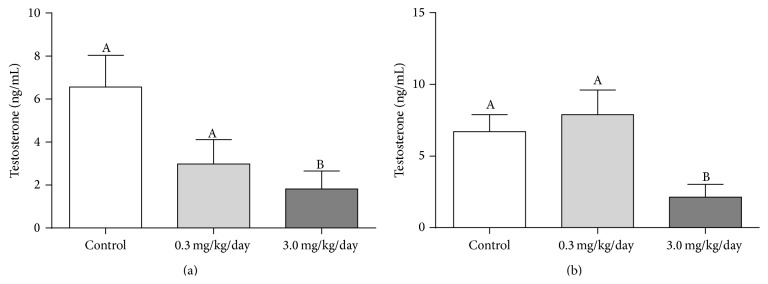
Serum testosterone levels of (a) 105-day-old animals (control (*n* = 9), 0.3 mg/Kg (*n* = 10), and 3.0 mg/Kg (*n* = 7)) and (b) 155-day-old animals (control (*n* = 9), 0.3 mg/Kg (*n* = 8), and 3.0 mg/Kg (*n* = 7)). Values expressed as mean ± SEM. One-way analysis of variance (ANOVA) test, followed by Tukey's test. Different letters indicate means that differ at 5% significance level.

At 105 days of age, arsenic blood levels of As_2_O_3_-treated groups (*μ*g/L, mean ± SEM, ANOVA followed by Tukey's test) were 16.02% and 109.94% higher than in the control group (*control, n* = 9: 9.05 ± 0.73; 0.3 mg/Kg/day, *n* = 10: 10.50 ± 1.17, *p* > 0.05; 3.0 mg/Kg/day, *n* = 9: 19.00 ± 2.18, *p* < 0.05). After treatment was suspended at the age of 155 days (*n* = 10 per group), blood arsenic concentration in the treated groups was comparable to the control group (data not shown). The hormonal dosages revealed that testosterone levels were diminished in the group treated with the highest dose, a condition that remained even after suspension of treatment (Figure 2).

**Figure 3:**
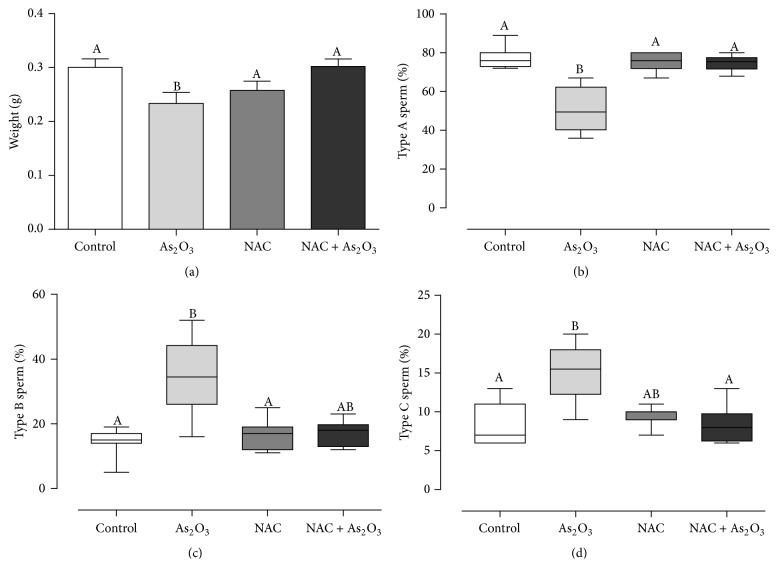
(a) Weight of full seminal vesicle (*n* = 8 per group). Values expressed as mean ± SEM. One-way analysis of variance (ANOVA) test, followed by Tukey's test. (b), (c), and (d) Sperm motility of controls (*n* = 7) and mice treated with 3.0 mg/Kg of As_2_O_3_ (*n* = 8), 40 mM of NAC (*n* = 7), or both As_2_O_3_ and NAC (*n* = 8). Values expressed as median. Kruskal-Wallis analysis of variance test, followed by Dunn's test. Different letters indicate mean or median that differs at the 5% significance level.

As previously shown in this study, treatment with As_2_O_3_ decreased absolute and relative weights of full seminal vesicle; however the weight was restored by the administration of NAC (Figure 3(a)). In the same manner, assessment of sperm motility revealed a decreased percentage of motile sperm and increased percentages of nonprogressive and immotile sperm in the As_2_O_3_-treated group; on the other hand, the group treated with both As_2_O_3_ and NAC showed lack of statistical difference in sperm motility when compared to the control group (Figures 3(b), 3(c), and 3(d)). As_2_O_3_ treatment also impaired DSP, as previously shown in this study. However, administration of NAC to As_2_O_3_-treated animals was able to restore sperm production, since this group presented DSP similar to controls (Table 3).

**Figure 4:**
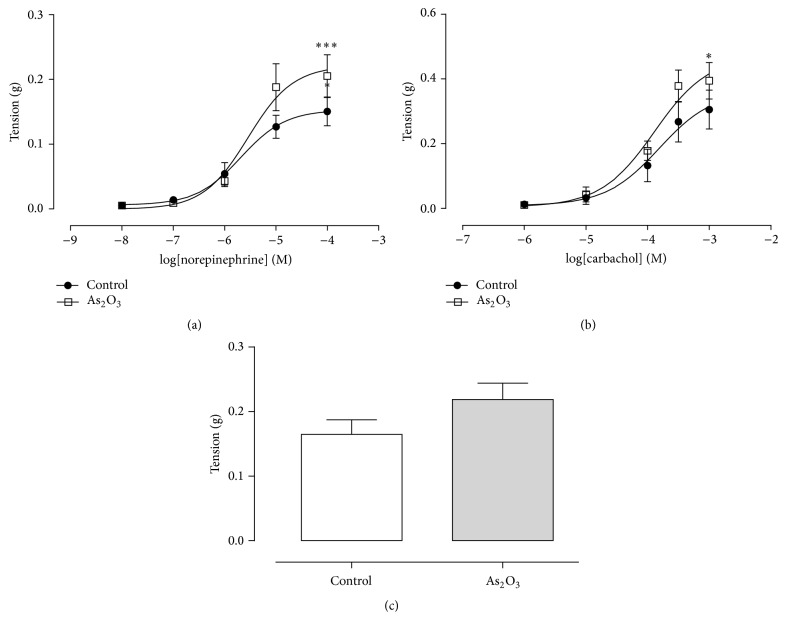
(a) NE (10^−8 ^M–10^−4 ^M) and (b) CCh (10^−6 ^M–10^−3 ^M) showing maximal tension (*E*_max_) and the potency (pEC_50_) in developing tension of CE duct of control group and animals treated with 3.0 mg/Kg/day of As_2_O_3_. (c) Tension developed by CE duct of control and As_2_O_3_-treated animals to 80 mM of KCl. Values expressed as mean ± SEM (*n* = 10 per group). One-way analysis of variance (ANOVA) test, followed by Tukey's test. ^*∗*^ *p* < 0.05; ^*∗∗∗*^ *p* < 0.001.

**Figure 5:**
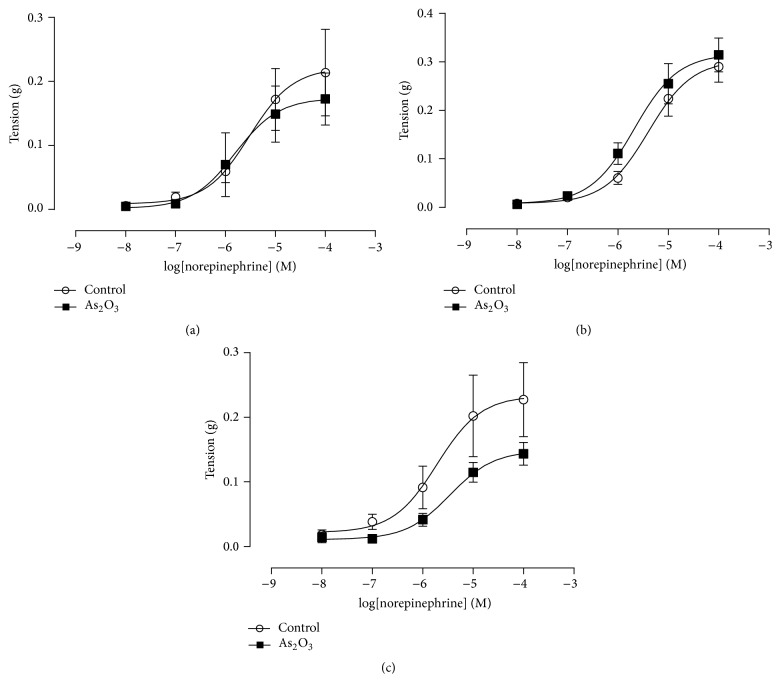
Concentration-response curve of CE duct to NE (10^−8^ M–10^−4^ M) after* in vitro* As_2_O_3_ exposure. (a) Control (*n* = 4) and exposed CE duct to 10 *μ*M of As_2_O_3_ (*n* = 6) for 45 minutes. (b) Control (*n* = 3) and exposed CE duct to 10 *μ*M of As_2_O_3_ (*n* = 4) for 2 hours. (c) Control (*n* = 7) and exposed CE duct to 100 *μ*M of As_2_O_3_ (*n* = 8) for 2 hours. Values expressed as mean ± SEM. One-way analysis of variance (ANOVA) test, followed by Tukey's test.

The tensions developed by NE or CCh are shown in Table 4. As noted, the pEC_50_ of NE- and CCh-induced tension of the epididymal duct were similar among groups. However, *E*_max_ of epididymal duct induced by NE and CCh was significantly elevated in the As_2_O_3_ group when compared to controls (Figures 4(a) and 4(b)). Furthermore, tension developed by 80 mM KCl was increased in this same group compared to control, but it was not significant (Figure 4(c)).* In vitro* incubation of epididymal duct with 10 or 100 *μ*M of As_2_O_3_ for 45 minutes or 2 hours did not change the potency or the maximal tension induced by NE (Figure 5).

## 4. Discussion

Results of the present study demonstrated that As_2_O_3_ exposure significantly decreased sperm quality and quantity as well as epididymal duct contractility. Treatment-induced adverse effects persisted 50 days beyond the end of treatment. Our results also revealed that the adverse effects of As_2_O_3_ were attenuated by cotreatment with the antioxidant NAC, showing that coadministration of antioxidants could minimize the adverse effects of As_2_O_3_ on the male reproductive tract and thus offer insight into potential pathways to mitigate the adverse effects in cancer patients.

Treatment significantly decreased sperm count and daily sperm production, seminal vesicle weight, and motility at both time points (at the end of treatment and 50 days after its suspension). Our findings are harmonious with results in mice treated with equivalent concentrations of As_2_O_3_ [10]. However, results of the present study expand on this earlier report by revealing that the adverse effects persist through 50 days after the treatments were stopped, a period that captures one complete spermatogenic cycle plus sperm transit through epididymis in the mouse.

In the present work, exposure to As_2_O_3_ reduced the daily sperm production, an effect that had long lasting effects since treated animals showed reduced daily sperm production after treatment discontinuation, suggesting that initial germ cells may be affected by the drug. When the male genital system is exposed to substances that increase ROS production beyond normal levels, such as heat, radiation, or chemicals, the spermatogenesis process may be impaired, since the testis represents one of the major organs placed at risk by exposure to agents that damage the genetic material [17]. Corroborating our findings, a study from 2009 demonstrated that exposure to As_2_O_3_ causes significant damage to DNA of primary spermatocytes [18].

We also demonstrated that As_2_O_3_-treated animals showed a reduced number of motile sperm even after the treatment had been discontinued. Considering that sperm motility is one of the most important parameters for assessing sperm quality and is highly correlated with fertile capacity [19], exposure to As_2_O_3_ can potentially decrease fertility in the treated animals. Because of the susceptibility of spermatozoa to oxidative stress caused by As_2_O_3_ and its vulnerability to lipid peroxidation, the fluidity of its lipid bilayer may be altered, which can compromise sperm motility and decrease sperm quality [20]. Furthermore, exposure to As_2_O_3_ is able to decrease mitochondrial membrane potential, which can also impair sperm motility capacity in a dose-dependent manner [10, 21].

The determination of reproductive organ weights is an important parameter for assessing the risk of toxicity in the male genital system [22]. Animals treated with As_2_O_3_ displayed no change in testis, epididymis, and prostate weights. However, we observed reduced weight of full seminal vesicle, a gland responsible for secretion of nutrients that compose the seminal fluid [23], which could indicate that secretion by epithelial cells is affected by treatment. Since this gland is androgen-dependent, it is possible that the decrease in testosterone levels, observed in the arsenic-exposed animals, contributed to impaired seminal secretion. One study suggested that ROS can damage Leydig cells, leading to decreased testosterone levels [24]. This effect was persistent even after the suspension of As_2_O_3_ treatment.

The measurement of circulating concentrations of arsenic revealed detectable levels even in control animals. We postulate that arsenic exposure in control animals likely arises from the presence of arsenic in rodent chow resulting from the addition of contaminated rice bran [25]. A small amount of arsenic is also found in drinking water [26]. As expected, As_2_O_3_-treated animals showed a dose-dependent augmentation of blood arsenic levels. However, since arsenic is rapidly metabolized and remains only a few hours in blood, it can accumulate in tissues of exposed individuals [27]. Thus, the measurement performed after the treatment suspension found that the blood arsenic levels were similar in all experimental groups, showing that the metal may have accumulated in the organs, contributing to the persistence of harmful effects.

We speculate that the mechanisms underlying the adverse effects of treatment on sperm parameters are increased ROS production in germ cells, since increased ROS levels have been previously related to As_2_O_3_ exposure [28–30]. ROS are generated continuously in small amounts in normal cells since they are essential for many biological functions [31, 32]; however, these inherent ROS levels are elevated in cancer cells, which become highly susceptible to apoptosis. As_2_O_3_ treatment of APL has shown efficacy due to increased ROS production in these cells, leading to oxidative stress that can trigger conformational changes that initiate the apoptotic cascade in leukemic cells [33]. Thus, the antitumor action of As_2_O_3_ has been linked to the induction of apoptosis of tumor cells [34]. Despite its effectiveness, exposure to As_2_O_3_ can cause apoptosis of normal cells, since chronic exposure, besides increasing ROS production, also results in reduced activity of enzymes of the antioxidant defense system such as superoxide dismutase, catalase, and glutathione, which are capable of scavenging excessive ROS, thereby keeping its level steady under physiological conditions. This imbalance between the pro- and antioxidant states of the cell can impair its physiology [35, 36], since the oxidative stress state causes oxidation of cell membrane proteins, enzymes, and DNA, leading to damage that can be irreversible [37]. The irreversibility of the effects may also be related to As_2_O_3_ excretion, since the complete elimination of inorganic arsenic from the body requires months [38].

Furthermore, As_2_O_3_ exposure may exhibit toxicity on other organs, since, after suspension of the treatment, animals showed increased liver and kidney relative weights which may be associated with hepatocellular hypertrophy and renal necrosis, measures that seem to be unrelated to body weight. Arsenic toxicity studies in laboratory animals showed that dose-related changes occur primarily in the liver and kidney, since the liver is the major metabolic site of inorganic As, whereas the kidney is the major pathway of excretion [39]. A recent work showed that a large amount of arsenic accumulates in these organs after exposure to As_2_O_3_, causing damage by ROS generation. Moreover, a significant decrease in the activity of scavenging enzymes such as catalase and glutathione peroxidase suggests a compromise of the antioxidant defense system and consequently a physiological effect on these organs [40].

Facing the constant risk of oxidative stress that germ cells are subjected to during treatment of APL with As_2_O_3_, which may impair reproductive physiology, the gonads may require antioxidant protection during gamete production. Thus, the use of substances with antioxidant properties has been proven effective at protecting germ cells against damage caused by ROS generation. In this sense, several studies have shown that NAC can attenuate the effects caused by oxidative stress [41, 42]. We therefore evaluated whether the antioxidant ability of NAC was able to restore the anti- and prooxidant balance of the cells and thus mitigate the harmful effects of As_2_O_3_ treatment. Indeed, when animals treated with As_2_O_3_ also received NAC in tap water, reproductive parameters previously affected by treatment such as daily sperm production, sperm motility, and seminal vesicle weight were similar to those of the control group, showing the protective effect of this antioxidant against oxidative stress damage. This is due to the fact that NAC is a precursor of glutathione (GSH), an important enzyme of the cellular antioxidant system that is able to stimulate and sustain its intracellular levels, which detoxify ROS. Furthermore, NAC has been shown to be effective in metal chelation [42, 43]. It is important to note that an antioxidant such as NAC can attenuate the adverse effects on the male reproductive tract probably without compromising effects on leukemia treatment, since a recent study showed that coadministration of As_2_O_3_ and resveratrol, an antioxidant obtained from grape, substantially amplified the anticancer effect of As_2_O_3_ *in vitro* [44]. Nevertheless more studies are needed to prove this hypothesis.

Results of the present study revealed, for the first time, decreased CE contractility in response to As_2_O_3_. The increase of the maximum CE duct response caused by* in vivo* treatment reflects the elevated contraction strength of the cauda epididymis muscle in response to As_2_O_3_ exposure. This increase occurred in the presence of both NE and CCh stimuli, suggesting that As_2_O_3_ acts on muscle cells; that is, it is not dependent on adrenergic and muscarinic receptors. It is known that changes in the contractile activity of these muscles that are capable of increasing or decreasing the sperm transit time can impair their maturation [45]. Florea and Büsselberg showed that As_2_O_3_ is involved in inhibiting growth and inducing apoptosis of malignant cells also by elevating intracellular calcium [46].* In vitro* treatment of CE duct with As_2_O_3_ for short periods (45 minutes to 2 hours) showed that this possible increase in intracellular calcium caused by the drug tends to be a slow process that only occurs with prolonged exposure, since there was no change in muscle contractility immediately after drug incubation.

In this work, compromised sperm quality may be due to the possible altered sperm transit time by epididymis, which is critical for sperm maturation, given sperm that leave the epididymis faster may present a reduced motility and fertility capacity. Previous results from our laboratory demonstrated that the decrease in sperm transit time through the cauda epididymis duct of rats is caused by increased contractility of smooth muscle surrounding the cauda, which compromises the fertile potential of these animals [47].

These findings, obtained in rodents, are important as they show reproductive implications for human health and provide data about reproductive toxicity of As_2_O_3_. We conclude, according to our objectives, that the chemotherapeutic agent As_2_O_3_ is toxic to the male mouse genital system, by compromising sperm quality and quantity, and that these effects are persistent even after suspension of the treatment. The compromised sperm quality in As_2_O_3_-exposed animals may be due to the altered epididymal duct contractility. Furthermore, the administration of the antioxidant NAC ameliorates the harmful effects of the drug on the male genital system.

## Supplementary Material

Supplementary Material shows the genital system of Swiss male mice. CE duct segment of approximately 1.0 cm length was isolated and attached to a force tranducer in order to record the isometric tension developed by the organ after in vitro or in vivo As_*2*_O_*3*_ exposure.

## Figures and Tables

**Table 1:** Full seminal vesicle, kidney, and liver weights at 105 and 155 days of age.

Parameters	Control (*n* = 10)	0.3 mg/Kg (*n* = 10)	3.0 mg/Kg (*n* = 10)
105-day-old mouse			
Body weight	40.36 ± 0.73	40.07 ± 1.06	41.15 ± 0.68
Absolute weight			
Full seminal vesicle (mg)	386.70 ± 22.55^a^	339.0 ± 15.66^a,b^	324.60 ± 11.54^b^
Relative weight			
Full seminal vesicle (mg/g)	9.62 ± 0.53^a^	8.56 ± 0.46^a,b^	7.84 ± 0.26^b^
Kidney (mg/g)	7.70 ± 0.26	8.01 ± 0.27	8.01 ± 0.23
Liver (mg/g)	55.68 ± 1.62	55.78 ± 0.56	57.89 ± 1.46
155-day-old mouse			
Body weight	39.02 ± 1.19	40.43 ± 1.55	39.96 ± 1.01
Absolute weight			
Full seminal vesicle (mg)	467.90 ± 24.41^a^	401.00 ± 28.27^a,b^	347.70 ± 27.67^b^
Relative weight			
Full seminal vesicle (mg/g)	10.89 ± 0.47^a^	9.68 ± 0.72^a,b^	8.52 ± 0.52^b^
Kidney (mg/g)	7.59 ± 0.27^a^	7.62 ± 0.18^a,b^	8.53 ± 0.38^b^
Liver (mg/g)	53.24 ± 1.47^a^	54.12 ± 1.53^a,b^	61.37 ± 2.70^b^

Values expressed as mean ± SEM. One-way analysis of variance (ANOVA) test, followed by Tukey's test.

Different letters indicate groups that differ at 5% significance level.

**Table 2:** Sperm counts of control and treated animals.

Parameters	Control (*n* = 10)	0.3 mg/kg (*n* = 10)	3.0 mg/kg (*n* = 10)
105-day-old mouse			
Sperm head count (×10^6^/testis)	22.53 ± 0.85^a^	21.12 ± 0.84^a,b^	16.11 ± 0.53^b^
Sperm head count (×10^6^/g testis)	218.70 ± 11.35^a^	205.50 ± 10.59^a,b^	161.70 ± 9.40^b^
Daily sperm production (×10^6^/testis)	4.65 ± 0.18^a^	4.36 ± 0.17^a,b^	3.32 ± 0.11^b^
Daily sperm production (×10^6^/g testis)	45.19 ± 0.18^a^	42.44 ± 2.19^a,b^	33.37 ± 1.95^b^
155-day-old mouse			
Sperm head count (×10^6^/testis)	22.11 ± 1.29^a^	23.67 ± 1.15^a,b^	14.13 ± 1.00^b^
Sperm head count (×10^6^/g testis)	242.8 ± 12.63	304.3 ± 26.53	241.3 ± 22.77
Daily sperm production (×10^6^/testis)	4.56 ± 0.27^a^	4.98 ± 0.23^a,b^	3.53 ± 0.21^b^
Daily sperm production (×10^6^/g testis)	50.11 ± 2.61	62.89 ± 5.57	49.73 ± 4.73

Values expressed as mean + SEM. One-way analysis of variance (ANOVA) test, followed by Tukey's test.

Different letters indicate groups that differ at 5% significance level.

**Table 3:** Sperm counts in controls and animals treated with 3.0 mg/Kg of As_2_O_3_, 40 mM of NAC, and both As_2_O_3_ and NAC.

Parameters	Control (*n* = 8)	As_2_O_3_ (*n* = 8)	NAC (*n* = 8)	NAC + As_2_O_3_ (*n* = 8)
Sperm head count (×10^6^/testis)	21.89 ± 1.06^a^	16.96 ± 0.71^b^	22.25 ± 1.24^a^	21.97 ± 1.29^a^
Sperm head count (×10^6^/g testis)	199.50 ± 6.55^a^	150.90 ± 9.25^b^	198.9 ± 7.09^a^	214.70 ± 12.85^a^
Daily sperm production (×10^6^/testis)	4.52 ± 0.22^a^	3.50 ± 0.15^b^	4.59 ± 0.26^a^	4.54 ± 0.26^a^
Daily sperm production (×10^6^/g testis)	41.22 ± 1.36^a^	31.08 ± 1.83^b^	41.06 ± 1.46^a^	44.34 ± 2.65^a^

Values expressed as mean + S.E.M. One-way analysis of variance (ANOVA) test, followed by Tukey test.

Different letters indicate groups that differ at 5% significance level.

**Table 4:** Potency (pEC_50_) and maximal tension (*E*_max⁡_) developed by NE and CCh on CE duct.

Parameters	Control (*n* = 8)	As_2_O_3_ (*n* = 8)
pEC_50_ NE	5.687 ± 0.237	5.544 ± 0.248
*E*_max⁡_ NE	0.1529 ± 0.0139^a^	0.2209 ± 0.0227^b^
pEC_50_ CCh	3.814 ± 0.341	3.890 ± 0.213
*E*_max⁡_ CCh	0.3621 ± 0.0744^a^	0.4687 ± 0.0573^b^

Values expressed as mean + S.E.M. One-way analysis of variance (ANOVA) test, followed by Tukey test.

Different letters indicate groups that differ at 5% significance level.
