# Supplementary material for: The Coadministration of N-Acetylcysteine Ameliorates the Effects of Arsenic Trioxide on the Male Mouse Genital System
Source: Oxid Med Cell Longev. 2015 Dec 29;2016:4257498. doi: 10.1155/2016/4257498 (PMC4709715; doi:10.1155/2016/4257498)
Supplement: Supplementary file 1 — Supplementary Material shows the genital system of Swiss male mice. CE duct segment of approximately 1.0 cm length was isolated and attached to a force tranducer in order to record the isometric tension developed by the organ after in vitro or in vivo As2O3 exposure. [file 4257498.f1.pdf]

SUPPLEMENTAL FIGURE LEGEND (color reproduction only on the Web)

Figure S1.

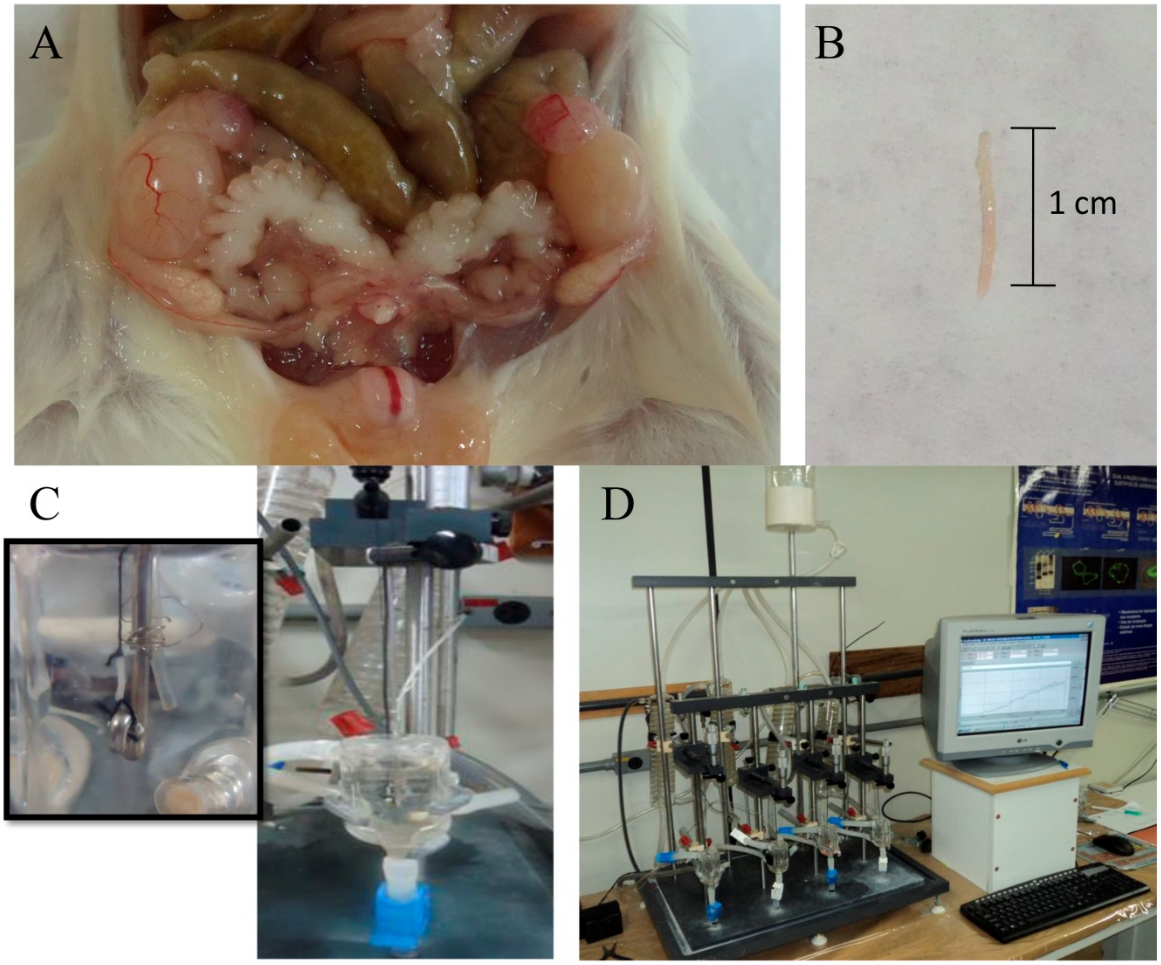

Figure S1. A) Genital system of Swiss male mice. B) CE duct segment of approximately 1.0cm length was isolated C) Segment was attached to FORT10 isometric force transducer (WPI, USA) connected to Transbridge 4M Transducer Amplifier (WPI, USA) to record the isometric tension development in a PC-based System (MP100, Biopac System INC., USA) and D) Data were analyzed off-line using the software AcqKnowledge version 3.5.7 (Biopac System INC., USA).
